# Solid Lipid Microparticles by Spray Congealing of Water/Oil Emulsion: An Effective/Versatile Loading Strategy for a Highly Soluble Drug

**DOI:** 10.3390/pharmaceutics14122805

**Published:** 2022-12-14

**Authors:** Alessandro Candiani, Andrea Milanesi, Andrea Foglio Bonda, Giada Diana, Elia Bari, Lorena Segale, Maria Luisa Torre, Lorella Giovannelli

**Affiliations:** 1 Department of Pharmaceutical Sciences, Università del Piemonte Orientale, Largo Donegani 2, 28100 Novara, Italy; 2 APTSol S.R.L., Largo Donegani 2, 28100 Novara, Italy; 3 Pharmaexceed S.R.L., Piazza Castello 19, 27100 Pavia, Italy

**Keywords:** spray congealing, solid lipid microparticles, microencapsulation, W/O emulsion

## Abstract

Spray congealing technique was exploited to produce solid lipid microparticles (SLMp) loaded with a highly water-soluble drug (metoclopramide hydrochloride) dissolved in the aqueous phase of a water in oil (W/O) emulsion. The use of an emulsion as starting material for a spray congealing treatment is not so frequent. Moreover, for this application, a W/O emulsion with a drug dissolved in water is a totally novel path. A ternary diagram was built to optimize the emulsion composition, a factorial design was used to identify the factors affecting the properties of the microparticles and a Design of Experiment strategy was applied to define the impact of process conditions and formulation variables on the SLMp properties. SLMp were characterized by particle size distribution, morphology, residual moisture, drug content, release behavior, FT-IR analysis and XRPD. The obtained microparticles presented a spherical shape, particle size distribution between 54–98 µm depending on atomizing pressure used during the production step and 2–5% residual moisture 4 days after the preparation. XRPD analysis revealed that lipid polymorphic transition alfa-beta occurs depending on the presence of water. In vitro drug release tests highlighted that all the formulations had a reduced release rate compared to the drug alone. These results suggest that spray congealing of a W/O emulsion could be proposed as a good strategy to obtain SLMp with a high loading of a hydrophilic drug and able to control its release rate.

## 1. Introduction

Particle engineering can offer many approaches to overcome the technological limitations that occur during the development of drug delivery systems. Among the most popular technologies used in the production of particle-based delivery systems (e.g., coacervation, solvent evaporation, ionic gelation, extrusion, spray drying, spray cooling and fluid bed hot melt coating), only some of these are adequate for the treatment of lipid formulations [1,2,3]. The use of lipid excipients in pharmaceutical products offers interesting benefits: their costs are relatively lower than those of other excipients (e.g., polymers) obtained through complex chemical processes. Moreover, lipid excipients are biodegradable and GRAS (generally recognized as safe) substances [4,5] that can be used in solvent-free processes and this makes them suitable for large-scale production [6].

Spray congealing and fluid bed hot melt coating are cost-effective and solvent-free techniques commonly adopted when lipid excipients are the principal components of the formulation [7,8]. In detail, spray congealing, also called spray chilling or cooling, consists of the atomization of a molten slurry, which is usually a solution or a suspension of an active pharmaceutical ingredient (API) in a melted carrier, into a chamber where the temperature is maintained below the carrier melting point [1,9]. When the droplets come in contact with the cold air, they immediately undergo a rapid solidification responsible for their transformation into solid microparticles (SLMp) [1].

According to the nature of the atomized slurry (solution, dispersion and, in rare cases, emulsion), SLMp with different structures and properties can be obtained. In the first case, the drug is dissolved into a molten carrier, forming a one-phase homogeneous system. During the solidification of the droplets, API can re-crystallize in its original form or a different one, can be entrapped into the final product in an amorphous form, or can be molecularly dispersed in the carrier, forming the so-called solid solution microparticles [2,10,11]. Wong et al. [12] prepared SLMp starting from an ibuprofen solution in cetyl alcohol or stearic acid. Tomšik et al. [13] used this approach to obtain Gelucire^®^ 53/10 microparticles loaded with wild garlic extract to improve the solubility of this poorly water-soluble compound. In the second case, when the starting point is the suspension of the drug in the molten carrier, there will be no possibility that the final product can be a solid solution. Suspension as starting material is usually proposed because it is suitable for synthetic, natural and biotechnological active substances. In this regard, Sorita et al. [14] successfully used the spray congealing technique for the production of curcumin-loaded microparticles; insulin-loaded microparticles were prepared by Maschke et al. [15] and lyophilized gonadorelin was incorporated in different triglycerides by Traub-Hoffmann et al. [16]. When SLMp are produced by spray congealing, the hydrophilic or lipophilic characteristics of the carrier excipient represent the main driving force that affects drug release behavior. Recently, an interesting and comprehensive review of spray congealing proposed a classification of the most adequate and used excipients [2]. The carriers can be divided into two classes: lipophilic matrix materials, such as triglycerides, fatty acids, fatty alcohols and waxes, and hydrophilic matrix materials (polyethylene glycols and poloxamers). They can be used alone or in combination, giving rise to SLMp with different drug release behaviors suitable for many fields of application. For example, moxidectin (a veterinary anthelmintic drug) was successfully formulated in an injectable sustained delivery system (able to provide 6 months of protection for dogs) by the use of the molten solution technique and the selection of a lipophilic matrix carrier [17,18,19]. Bertoni et al. [20] suspended three BCS class II drugs (carbamazepine, tolbutamide and cinnarizine) in molten Gelucire^®^ and submitted the mixtures to spray congealing to investigate the influence of the API properties on the characteristics of semi-crystalline solid dispersion based on this excipient. They obtained spherical and non-aggregated SLMp with quite a smooth surface and demonstrated that the drug solubility was mainly influenced by the carrier, while the drug release behavior depended on the API solid state in the microparticles. In another work, a lipophilic matrix excipient (Compritol 888 ATO, a glyceryl behenate derivate) was used to suspend a highly water-soluble drug (theophylline) to obtain controlled drug-release microparticles [21]. Tristearin, stearic acid or glyceryl behenate were used to encapsulate salbutamol sulphate [22], while a mixture of microcrystalline wax and stearyl alcohol was selected as carrier material for verapamil hydrochloride loaded controlled release microparticles [23].

Based on the previous statements, it is clear that the use of a suspension is the most common approach when the spray congealing process is carried out because it is more flexible and easier to develop than when a solution is the starting point (i.e., it is not always possible to identify a lipid mixture able to solubilize a specific drug); moreover, the choice of the proper excipients can manage the API release. Conversely, using an emulsion as starting material for a spray congealing treatment is not so frequent; the literature offers only a few examples [6,24,25]. In this case, a water-in-oil emulsion, in which the oily phase is constituted by the melted lipid and the aqueous phase is an aqueous solution or a hydrophilic molten excipient in which the API could be solubilized, is prepared and submitted to the spray congealing process. Chambi et al. [24] explored this approach to encapsulate hydrophilic compounds of different molecular weights (glucose, casein and hydrolyzed casein) in stearic acid. Another protein hydrolysate (soybean) was encapsulated by Salvim et al. [25] in partially hydrogenated cottonseed oil. More recently, Bertoni et al. [6] described the spray congealing of a water-in-oil (W/O) emulsion loaded with a water-soluble dye (rhodamine), in which the “aqueous phase” was a hydrophilic molten excipient (PEG 400), and the “oily phase” was a long chain glyceride.

The aim of this research was the production of SLMp by spray congealing starting from a W/O emulsion. Metoclopramide hydrochloride, a highly water-soluble drug selected as the model drug, was dissolved in the aqueous phase of the emulsion. A ternary diagram was built to stabilize the emulsion composition and a factorial design was used to understand the key factors affecting microparticle properties. SLMp were thoroughly characterized, and the obtained results were compared to those of SLMp produced starting from a suspension to evidence similarities and differences.

## 2. Materials and Methods

### 2.1. Materials

Metoclopramide hydrochloride (MCP, batch 100220002) was purchased from AMSA (Milan, Italy) and used “as received”. Softisan 154 (SFT, hydrogenated palm oil, batch 001296) and Imwitor 600 (IMW, polyglyceryl-3 poly-ricinoleate, batch 003153) were kindly donated by IoI Oleo GmbH (Witten, Germany). All other chemicals were of analytical grade.

### 2.2. Formulation of the Emulsions

**Figure 1:**
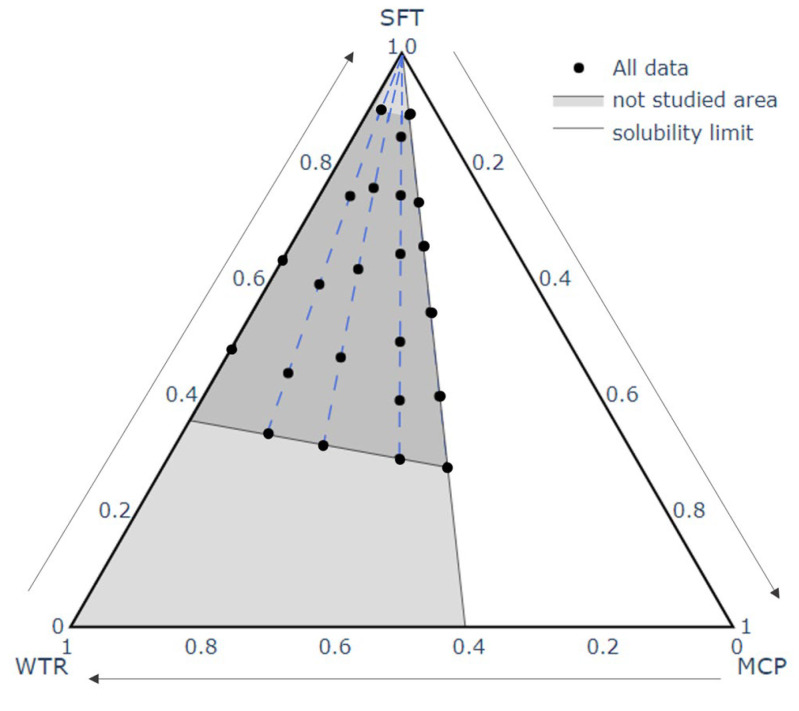
Experimental mixture design of the formulation study. WTR = Water, MCP = Metoclopramide hydrochloride, SFT = Softisan. The experimental points are shown as black points and are arranged in the studied area (in grey) following different MCP/WRT ratios (1.5, 1, 0.5, 0.25, and 0) represented by the blue dashed lines. The white area represents the water solubility limit of MCP.

Different emulsions usable as a melted slurry in the spray congealing process were prepared and their composition was defined by a mixture design approach to planning the proportions of the components. Three components, MCP, water (WTR) and Softisan 154 (SFT), were varied from 0 (=0%) to 1 (=100%) and a constraint was set to avoid exceeding the maximum water solubility of the drug (1.5 g/mL). The experimental area resulted in a triangle having the following coordinates MCP:WTR:SFT = 0:0:1, 0:1:0, and 0.6:0.4:0 (Figure 1).

Each emulsion was prepared in a flat bottom test tube of 30 mm height: firstly, SFT was melted and maintained at a temperature of about 15 °C above its melting point. Then, IMW (the emulsifier) in a concentration equivalent to 2% of water was solubilized in the molten lipid under magnetic stirring. Next, the drug was dissolved in water, the obtained solution was added to the lipid phase and the test tube was sealed. Each emulsion was kept under magnetic stirring for about 30 min, left to rest on the heating plate for about 1 h and then visually inspected to evaluate its stability and processability. The composition of the emulsions was defined referring to the mixture design and according to the following scheme: at first different proportions between MCP and water (i.e., MCP/water ratio) were set (1.5, 1, 0.5, 0.25, and 0) and the corresponding lines from the vertex of the domain (MCP:WTR:SFT = 0:0:1) were drawn. In all the formulations tested, the weight ratio between IMW and WTR was kept constant (2%). Starting from the vertex and following the proportion lines, different formulations were produced to find those able to be stable during a predefined time interval. Finally, the emulsion stability was evaluated by visual inspection; the results were collected and plotted on a ternary diagram defining the limits and the areas within which the emulsions could be considered adequate to be processed by spray congealing.

### 2.3. Preparation of Microparticles

Microparticles were produced by spray-congealing technology using a Buchi Mini Spray Dryer B-290 equipped with a spray-congealing accessory. The starting molten slurries, the emulsions with a composition derived from the mixture design, were prepared as described above, increasing the batch size from a test tube size to about 20 g. The spray congealing conditions were the same for all the formulations: a nozzle with a 2.0 mm tip hole and 2.8 mm cap hole, thermostated with a thermic oil at 125 °C, was used. The molten slurry was led to the atomizing nozzle by the peristaltic pump and sprayed into the tower in which the airflow was cooled down at 9 °C by a dehumidifier (Buchi dehumidifier B-296^®^). Finally, the solid microparticles were recovered from the collecting vessel at the bottom of the cooling chamber and stored in a polypropylene closed falcon tube at room temperature.

### 2.4. Factorial Design

A Design of Experiment (DoE) strategy was applied to investigate the impact of some spray congealing process conditions and formulation variables on the final product characteristics. In detail, different inputs, such as the percentage of drug loading, the ratio between the API and water (API:H_2_O) and the atomization pressure, were evaluated. Therefore, a Factorial design with three factors, two of which with three levels (API percentage: 7.5%, 12.5% or 22.5%; and API:H_2_O ratio: 1:1; 1:1.5 or 1.5:1) and one with two levels (Pressure Nozzle: 0.35 or 1.75 bar) was applied and 18 experimental points were generated (Table 1).

The randomization and statistical analysis of the experimental points were performed using Design-Expert^®^ v12 software (State-Ease Inc., Minneapolis, MN, USA). To select the best model a sequential sum of squares for the 2-factorial interaction (2FI) has been applied for *p*-value < 0.1.

The randomization of the experimental points and the analysis of the DoE were performed utilizing Design-Expert^®^ Software (Version 13, State-Ease Inc., Minneapolis, MN, USA). As a reference, other microparticles were produced using a suspension of API in the melted lipid excipient as a starting material. In detail, three suspensions characterized by three different MCP loadings (8, 13, and 28% *w*/*w*) were submitted to spray congealing treatment and the obtained final products were characterized to compare their properties and behavior to those of the systems obtained starting from an emulsion. Moreover, drug-unloaded microparticles, composed only of lipid phase, and physical mixtures (Phy mix) of excipients and API aqueous solution or excipients and API alone (in the same weight ratio of the SLMp) were prepared and used for comparison. All the analyses were performed on microparticles immediately after preparation and after 4 days of open-air storage.

### 2.5. Characterization of Microparticles

#### 2.5.1. Particle Size Distribution

The microparticle size distribution was evaluated by a laser particle size analyzer (Bettersizer 2600, Bettersize Instruments, Munich, Germany) with dry dispersion model equipment (BT-903). Results are reported as D10, D50 and D90 values and are the average of three determinations.

#### 2.5.2. Morphological Analysis

The morphology of drug powder and microparticles produced were observed by an optical stereomicroscope (Stereomicroscope Leica S9i, Buccinasco, Milano, Italy) and their shape regularity was defined according to the value assumed by the shape factor calculated using an image analysis software (ImageJ software, National Institute of Health, Bethesda, MD, USA). To investigate in detail the surface of microparticles, some samples were characterized by scanning electron microscopy (SEM, Phenom XL, Thermo Fischer Scientific, Waltham, MA, USA) using 15 kV voltage, 0.1 Pa vacuum pressure, backscattering, and secondary electrons detectors. Samples were manually dispersed over stubs with carbon stickers and metal covered with gold by a sputter-coating process to improve image quality. Zoom and measurement scales were variable and reported in each image.

#### 2.5.3. Determination of the Residual Moisture Content

Water content was determined on freshly prepared microparticles (t0) and microparticles after 4 days of open-air storage (t4) by using coulometric Karl Fischer titration (HI 904 Karl Fischer Coulometric Titrator, Hanna Instruments, Woonsocket, RI, USA). Samples were placed into a vial with dichloromethane/methanol (DCM/MeOH ratio: 1/1) and sonicated for 15 min to accelerate their dissolution. Then, a precisely weighed aliquot of the obtained solution was withdrawn by a syringe and immediately inserted into the titration vessel. Results are reported as the percentage of residual water recovered (% *w*/*w*). The dichloromethane/methanol mixture (DCM/MeOH ratio: 1/1) was used as blank. All the analyses were performed at least in triplicate. Water recovery at t0 Equation (1) and water loss percentage after 4 days (Equation (2)) were calculated as reported below:

Water recovery (WTR 0 %) = water content t0 (%)/water amount in formulation (%) × 100(1)

Water loss (WTRl %) = water content t4 (%)/water content t0 (%) × 100(2)

#### 2.5.4. Determination of Drug Content

A precisely weighed quantity of microparticles was added to 500 mL of phosphate buffer solution (PBS) (0.1 M, pH = 6.8) which was held under magnetic stirring and heated up to 70 °C to allow the melting of the carrier, facilitating the complete solubilization of the loaded drug. Next, 5 mL of solution was withdrawn, cooled down at room temperature, filtered through a 0.22 μm filter and analyzed spectrophotometrically at 273 nm (Beckman Coulter, DU 730 Spectrophotometer). The drug concentration and, as a consequence, the drug amount (mg) in the sample was determined according to the experimental calibration curve (y = 37.041x + 0.0106; linearity curve in the range of 0.5–50 × 10^−2^ mg/mL, R^2^ = 0.9998), using PBS 0.1 M (pH 6.8) as blank. Each sample was analyzed in triplicate. The drug content (%) was calculated using the following Equation (3):

Drug content (%) = experimental drug amount (mg)/microparticles weighed (mg) × 100(3)

Theoretical drug content was calculated on the preparation after 4 days of storage with the following Equation (4):

Theoretical MCP title = MCP(g)/MCP + SFT+ IMW(g) × 100(4)

The Encapsulation Efficiency was calculated according to Equation (5):

Encapsulation Efficiency (EE %) = Drug content (%)/Theoretical title on absolute dry (%) × 100(5)

#### 2.5.5. In Vitro Drug Release Tests

In vitro drug release profiles of microparticles were constructed to evaluate the amount of MCP released by the different batches of microparticles in phosphate buffer solution (PBS) 0.1 M (pH 6.8) over time. Briefly, a precisely weighed amount of the different SLMp was put in 500 mL PBS 0.1 M (pH 6.8) and maintained under continuous stirring (300–500 rpm). Then, at different time intervals (2, 5, 10, 15, 20, 30, 60, 120, 180, 240 and 1440 min), an aliquot of solution (5 mL) was withdrawn without replacement, filtered through a 0.22 μm filter and analyzed by UV-Vis spectrophotometer at 273 nm wavelength (Beckman Coulter, DU 730 Spectrophotometer). The percentage of drug released after each time was calculated considering the initial MCP content. As a reference, the same test was carried out on pure MCP HCl and the excipient mixture (SFT and IMW). The results are the average of three determinations.

#### 2.5.6. Fourier Transform-Infrared Spectra (FT-IR) Analysis

Infrared spectra of drug-loaded microparticles, drug-unloaded microparticles and pure drug were performed by an Alpha II FT-IR spectrometer with platinum Attenuated Total internal Reflectance (ATR) module (Bruker, Rosenheim, Germany). The spectrometer runs Opus 7.8 software.

#### 2.5.7. X-ray Powder Diffraction (XRPD)

Selected samples (drug-loaded microparticles, placebo microparticles, drug-excipients physical mixture, SFT after spray congealing treatment and drug alone) were submitted to X-ray analysis to evaluate their crystalline structure. X-ray Powder Diffraction (XRPD) spectra were recorded on an APD 2000 Pro GNR diffractometer at room temperature, using a CuKα tube (40 kV, 30 mA, λ = 1.5418 Å) as an X-ray source and scintillator as detector type. Data collection was made in 2θ step scan mode, at a scan speed of 0.04°/s in the range of 3° to 40° 2θ and from 1.5 to 20 at a scan speed of 0.02°/s.

## 3. Results and Discussion

### 3.1. Formulation of the Emulsions

**Figure 2:**
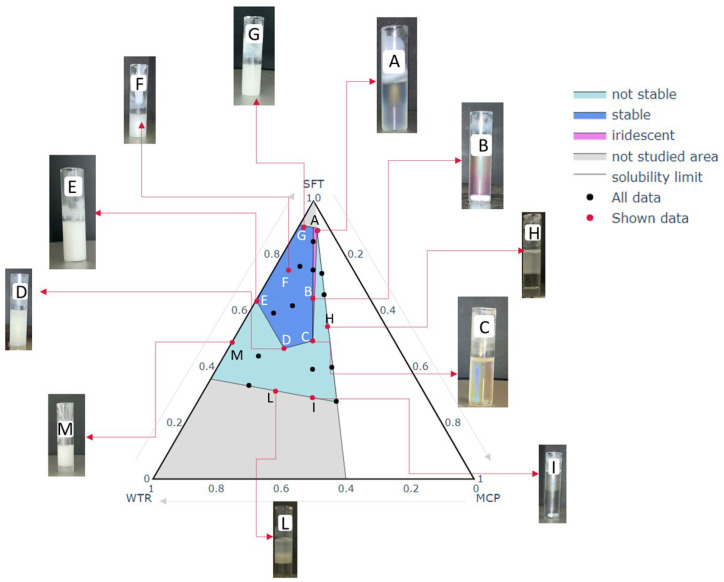
Stability and visual aspect of the emulsions at 75–80 °C. Three different appearances of the emulsions were identified: stable, iridescent and not stable, respectively represented by the blue, fuchsia and turquoise area. For each of these areas, some experimental point images are reported (red points).

When an emulsion has to be processed via spray congealing, it is mandatory to be characterized by adequate stability because a stable molten slurry avoids phase separation in the feeding line (from beaker to nozzle) and increases the possibility of obtaining a final product which is uniform in composition. Considering the novelty of applying the spray congealing technique to a W/O emulsion, a formulation study was indispensable to understand the behavior of the different emulsions under treatment but, above all, to guarantee the availability of a starting slurry that can be successfully processed to obtain the desired final SLMp. After a preliminary excipient selection, a mixture design approach was adopted as the key to understanding the best proportions of lipid, MCP and water to obtain a stable emulsion. As shown in Figure 2, points A, B, C, D, E and G delimit an area in which stable emulsions were obtained. Outside this area, all the formulations were liable to phase separation even if with different timing (area marked color in Figure 2).

Nevertheless, inside the stable emulsion area, not all the formulations had the same behavior; in detail, it can be noted that there was a narrow portion between the vertex and the points A, B and C, where the emulsions assumed an iridescent color remaining transparent, differently from the other stable emulsions that always had a milky white and opaque appearance. The transparency of some formulations can be attributed to a smaller size of the drops of their dispersed phase compared to those of the cloudy emulsions, hypothesizing that watery droplets at narrow nanoscale dimensions were generated. This is supported by other authors that obtained oil-in-water nano-emulsions characterized by a translucent appearance [26] or developed lipid-based microparticles by spray congealing starting from a W/O emulsion characterized by nano-sized droplets of the hydrophilic phase (PEG 400 and a pink dye) [6].

In the case of MCP emulsions, the interesting result is represented by the relatively small area associable with the nano-systems compared to the area corresponding to a cloudy stable emulsion. Furthermore, this narrow area partially overlaps the MCP/WTR ratio line that corresponds to a value of 1, meaning that only the proportion of 50:50 MCP:WTR leads to obtaining such nanoscale systems. This fact confirms that, for interfacial stability equilibrium, both the excipients and API play a relevant role and that all the mixture’s components contribute to guaranteeing a stabilizing effect. The stabilizer content employed did not seem to have an effect on the quality/stability/color of the emulsions. These results allowed the identification of the area in which the experimental points would have been produced.

### 3.2. Particle Size Distribution (PSD)

The dimensions of microparticles can influence drug delivery rate, mouthfeel, shelf life, hygroscopicity, sedimentation rate in dispersion, etc. In addition, they could also significantly impact several downstream processes, such as dosing, transport, coating and agglomeration. Therefore, the characterization of the particle size distribution of the obtained microparticulate systems is critical to suppose or predict some of the abovementioned properties. The particle size distribution of the drug-loaded microparticles is reported in Table 2: the systems produced with 1.75 bar of atomizing pressure were characterized by a mean D90 of 54.34 ± 14.45 μm (mean of all the odd experimental points), and the microparticles produced with 0.35 bar of atomizing pressure by D90 of 97.37 ± 15.95 μm (mean of all the even experimental points). Suspension data are reported as a comparison.

**Figure 3:**
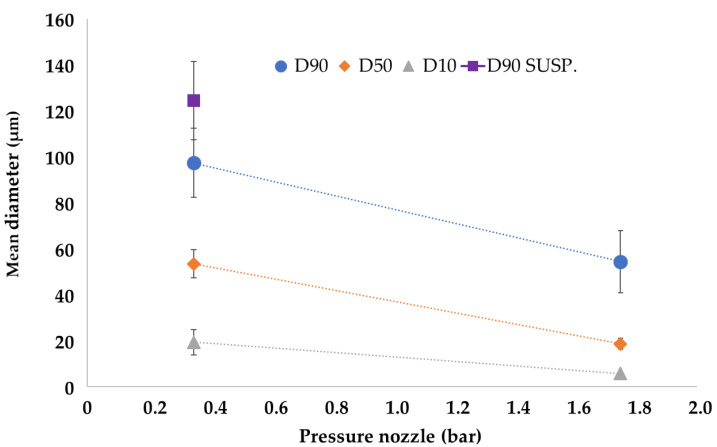
Effect plot of Pressure Nozzle process variable on SLMp particle size (error bars as Standard deviation, *n* = 3).

In general, it is evident that microparticles obtained with high atomizing pressure (EXPs. odd) were smaller than those produced using low pressures (EXPs. even). Furthermore, the effect of this process parameter is also evident considering D10 and D50, as shown in Figure 3, which reports the effect plot of the input variable “Pressure Nozzle” on the three output variables that describe the particle size (D10, D50 and D90).

The results agree with those reported in the literature [8,15]: high atomizing pressures reduced droplet size and, consequently, were responsible for a small microparticle diameter. However, it is noted that SLMp obtained when the starting material was a suspension were larger in diameter and this could be attributed to the presence of drug particles that occupied a larger volume than the emulsion drops. The strong effect of the Pressure Nozzle on particle size is also evident in factorial analysis, where the calculated model for D50 gave an R^2^ = 0.93 value using only the terms Intercept and Pressure Nozzle (*p*-value < 0.0001).

### 3.3. Morphological Analysis

**Figure 4:**
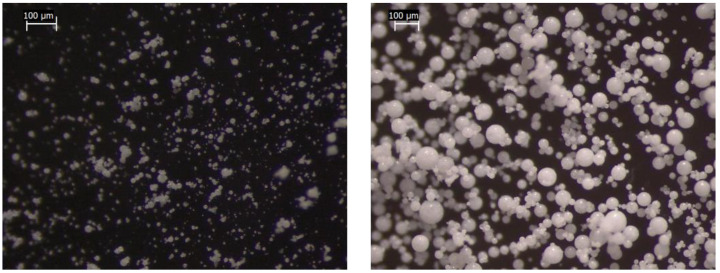
Optical microscopy images of microparticles prepared with 1.75 bar (EXP.3—(**left**)) and 0.35 bar (EXP.4—(**right**)) of atomizing pressure. Scale bar: 100 μm.

The stereomicroscope images of microparticles show that all the batches were composed of solid spherical units, proving that the spray congealing technique was suitable for obtaining regular in-shape particles. No differences could be appreciated between the formulations except for the particle size, which increased in those obtained with 0.35 bar of atomizing pressure (Figure 4).

**Figure 5:**
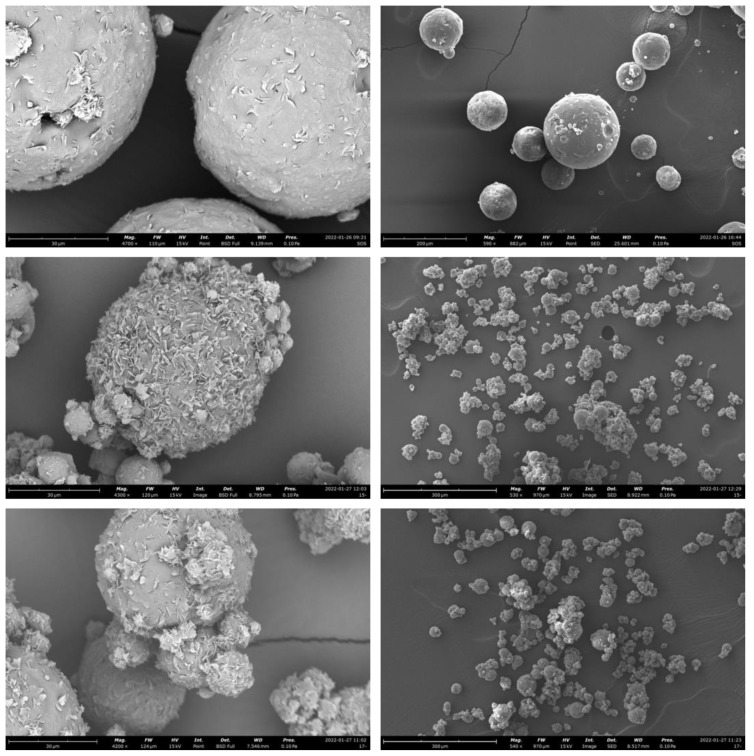
SEM images of microparticles obtained starting from a suspension with 12.5% of drug (**top**), EXP 5 (**middle**), EXP 17 (**bottom**), with different magnifications: 4200× (**left**) and 540× (**right**). Scale bar: 30 μm (**left**) and 300 μm (**right**).

The morphology of microparticles can be evaluated in detail by SEM images: their external surface was more or less “jagged”, with the absence of clearly defined pores. Water-free formulations (in which the drug was suspended in the lipid matrix) had a smoother surface than those produced starting from an emulsion, suggesting a role of water in determining the rough surface during the solidification of lipidic droplets (Figure 5). This hypothesis is supported by the literature: a smooth surface was typical of microparticles obtained using the suspension method [8], while Chambi et al. [24] prepared systems with a more jagged, porous and irregular surface spraying a W/O emulsion slurry. Moreover, in Salvim et al. [25], the emulsion technique used to encapsulate soybean proteins gave rise to microparticles with many pores and cavities on their surface.

No drug crystals were visible on microparticles surfaces, suggesting that MCP could be uniformly well dispersed in the matrix and that the rapid solidification of the system during the production step could be responsible for fast recrystallization resulting in the absence of large crystals or even for the no-crystal formation [27].

### 3.4. Determination of the Residual Water Content

Residual water content was determined by coulometric Karl Fischer titration on freshly prepared (t0) and 4 days-aged (t4) microparticles (Table 2). In general, microparticles with a higher amount of water in the starting formulation presented a higher residual water content at t0 (EXP 1–6), which ranged from 5% to 16%. However, at t0, the residual water was always lower than the initial water content. This event can probably be attributable to the production technique that involved heating during the emulsion preparation step and before the treatment of the formulation with spray congealing. Probably, a part of the water included in the composition of the emulsions evaporated, reducing the total water content of the final product at t0. No correlation between the process water loss and other variables was found.

**Figure 6:**
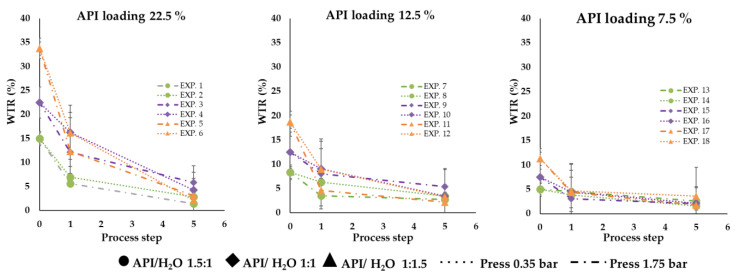
Water loss of formulations as a function of process steps.

Regardless of the amount of water present at t0, all the preparations showed water loss after the storage period of 4 days. In detail, the residual water present in all the systems 4 days after the preparation did not exceed 5% (i.e., between 2 and 5%), meaning that the water amount lost is significant. The percentage of water loss was higher in the case of EXP1, EXP2, EXP3, EXP4, EXP5 and EXP6 compared to the others and varied from 52% to 87%. For all the other preparations (EXP 7–18), the percentage of water loss was between 22% and 63%, with no significant differences between the subsets of microparticles presenting different drug content (EXP 7–12 vs. EXP 13–18). The water loss indicates that the system structure allowed a certain degree of internal/external gas exchange, leading the water to evaporate, get out of the particles and probably reach an equilibrium with the surrounding environment. The correlation between WTR t0 and water loss percentage was weak (R^2^ = 0.41) and no correlation between WTR t0 and WTR t4 was noticeable, suggesting that other factors influenced water retention. Figure 6 shows the water amount of each formulation during the three process steps (before spray = 0, after spray process = 1 and after 4 days of storage = 5); it is possible to observe how the water release rate is fast as much water is present inside the particle.

A correlation (R^2^ = 0.82) was found between drug content and Wt0, suggesting that an increase in API amount led to an increase in water entrapped into the microparticle structure. On the contrary, no correlation was found between API loading and Wt4. Considering that in the factorial design the variables API and API:H_2_O ratio were used as factors, the dependency of the water amount at t0 with the amount of MCP was generated by the design itself. For this reason, factorial analysis on Wt0 as a response variable was not performed.

### 3.5. Determination of Drug Content

The drug content assay showed consistent data and reflected the theoretical ones. EXP 1–6 presented the highest experimental drug content, EXP 13–18 the lowest and EXP 7–12 a drug loading included between the two other subsets of data. In the case of the first set of experiments (EXP 1–6), an appreciable variation in drug content was evident. In detail, experiments with API:H_2_O ratio 1.5:1 (EXP 1–2) had a lower drug content than experiments with API:H_2_O ratio 1:1 (EXP 3–4), while experiments with API:H_2_O ratio 1:1 (EXP 3–4) had, in turn, a lower drug content than experiments with API: H_2_O ratio 1:1.5 (EXP 5–6). This result can be attributable to the water loss process: microparticles with the highest water amount (API:H_2_O 1:1.5) lost most of the water after 4 days of storage, and this event was responsible for an increase in the percentage of drug content. For this reason, the API recovery on absolute dry formulation was calculated to reduce or eliminate the water effect on API assay evaluation. The results showed that API recovery on absolute dry formulation ranged from 92% to 107% (Table 2), indicating that the encapsulation efficiency for all 18 experimental points was good and suggesting the reliability and efficacy of the spray chilling technique for microencapsulation of a water-soluble drug using the emulsion technique. These results agree with the data reported in the literature: for example, Salvim et al. [24] encapsulated soybean proteins by the same production method, obtaining an incorporation efficiency of 96%.

These data are more interesting and relevant when compared to the encapsulation efficiency of microparticles obtained by the suspension technique. For the formulations containing 7.5, 12.5 and 22.5% of MCP in suspension into the lipid matrix, the encapsulation efficiency was 81.5, 79.9 and 47.6, respectively; the high percentages of drug affect the suspension stability reducing the encapsulation efficiency.

### 3.6. In Vitro Drug Release Study

**Figure 7:**
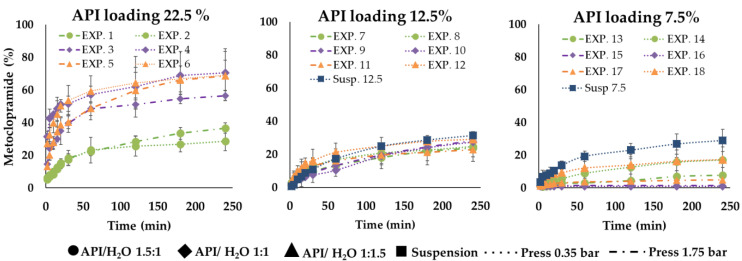
In vitro release profiles of experiments with the same API amount ((**left**) 22.5%, (**center**) 12.5% and (**right**) 7.5%).

**Figure 8:**
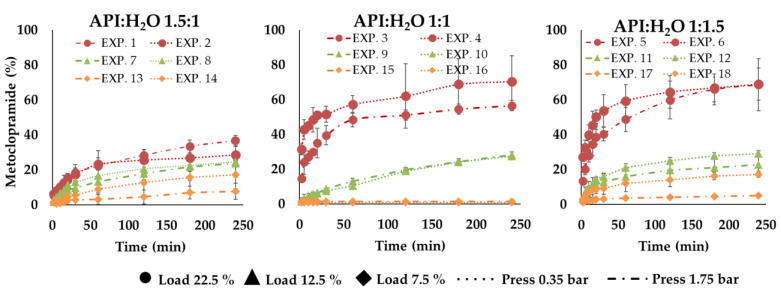
In vitro release profiles of experiments with the same API:H_2_O ratio ((**left**) 1.5:1, (**center**) 1:1, (**right**) 1:1.5).

**Figure 9:**
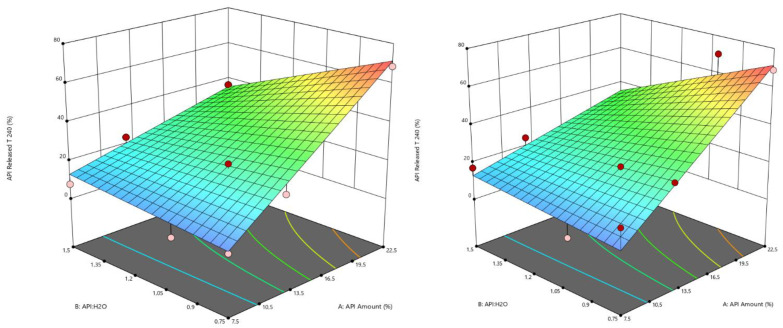
Contour plot of the API amount released at t240 (Left Pressure Nozzle = 1.75 bar, Right Pressure Nozzle = 0.35 bar).

The results of the in vitro drug release tests reveal that all the formulations had a reduced release rate compared to API alone, which solubilized immediately (in about 2 min) in the watery medium. In detail, within the first 5 min, microparticles from EXP1 to EXP6 showed a faster drug release compared to the other ones: for these six formulations, after 5 min from the beginning of the tests, the drug in solution was always over 6% (calculated on the experimental title) reaching even 42% only in the case of EXP 4. For the other samples, after the same time, the drug released did not exceed 6%; in some cases, it was less than 2%. After 60 min, EXP 1–6 released between 22% and 59% of the loaded drug, EXP 7–12 between 10% and 21% and EXP 13–18 between 1% and 12%. After 240 min (t240), these percentages were between 28% and 70% for EXP 1–6, between 22 and 29% for EXP 7–12 and between 1% and 17% in the case of EXP 13–18 (Figure 7, Figure 8, Figure 9, Figures S1 and S2 as additional documentations). No differences seem to be due to particle sizes.

The rate of the drug release process depended on the drug loading of microparticles and this was higher for the formulations with higher drug loading (22.5%). EXP 1 and 2 were characterized by a slower drug release rate than EXP 3–6. This behavior can be clearly noticed in Figure 7 and it can be attributable to the different API:H_2_O ratio (Figure 8). In general, SLMp can preserve the integrity of their structure in aqueous media without enzymes, so the drug release was controlled according to a diffusion mechanism. In particular, in this kind of system, drug diffusion occurs through pores and cavities that form little by little when the drug dissolves and goes out of the system [28]. Based on this statement, it is clear that the drug release performance of a particle structure, that embedded a small amount of a water-soluble API, can be different from that of a system loaded with an important drug dose (e.g., 7.5% vs. 22.5% loading).

As Albertini et al. [28] reported, even the presence of a surfactant or hydrophilic liquid cores in SLMp can improve their drug release behaviors. The obtained results on MCP SLMp evidence that the amount of water used in the different formulations affected the drug release and played an important role in defining the process rate. In detail, comparing the experimental points characterized by the same initial drug loading (22.5%), it can be observed that microparticles with API:H_2_O ratio 1:1.5 (EXP 5, EXP 6) and 1:1 (EXP 3, EXP 4) showed a faster drug release than those with API:H_2_O ratio 1.5:1 (EXP 1, EXP 2), probably because the imbalance towards water favors a faster release. Nevertheless, this effect was less evident when formulations with low drug content (EXP 7–18) were considered (Figure 7) because, in these cases, the drug release curves flatten out so that it is pretty hard to distinguish a definite and significant difference among them. This finding suggests that the amount of water in the molten slurry affected the increase of the API release; probably, when the water initially entrapped in the lipid matrix evaporated, it generated a structure with cavities that increased by increasing the starting water amount in the formulation, able to promote the dissolution media uptake into the systems and the diffusion of the drug solution in the bulk of the fluid. Unexpectedly, no significant differences were observed between the drug release performance of microparticles produced with different pressures, indicating that, in this case, the effect of particle size on the drug release behavior was overcome by the effect of the formulation composition.

The factorial analysis of variable t240 confirmed drug loading and the water amount effect on drug release, i.e., the amount of API released after 240 min. A significative model with R^2^ = 0.94 and a predicted R² = 0.89 was obtained using Equation (6); the corresponding contour plots are shown in Figure 9, while the estimations of the coefficients are available as additional documentation (Table S1).

t240 = b_0 + b_1 API Amount + b_2 API:H_2_O + b_3 API Amount × API:H_2_O (6)

At the end of the tests, for most of the preparations, the percentages of the drug released did not reach 100%. To understand if this result was linked to an analytical problem or to the inability of MCP to emerge from the lipid matrix structure, the release medium was heated above the lipid melting point, facilitating the drug’s dissolution eventually, still entrapped in the microparticle structure. After this treatment, the total amount of drug loaded into microparticles dissolved, confirming that the lipid structure of microparticulate systems was responsible for the limitation of total drug dose release (Figure S3, additional documentation).

Comparing the results of the in vitro drug release tests carried out on microparticles obtained starting from an emulsion to those prepared using a suspension, there were no differences if MCP loading was set at 12.5%, while the release rate was lower for the systems prepared with an emulsion if the drug content was 7.5%. The highest loading level (22.5%) could not be compared because of the impossibility of producing microparticles using the suspension method. Using a W/O emulsion instead of the suspension allowed particles with a higher drug loading and an API release tunable using different API:H_2_O ratios.

### 3.7. Fourier Transform-Infrared Spectra (FT-IR) Analysis

**Figure 10:**
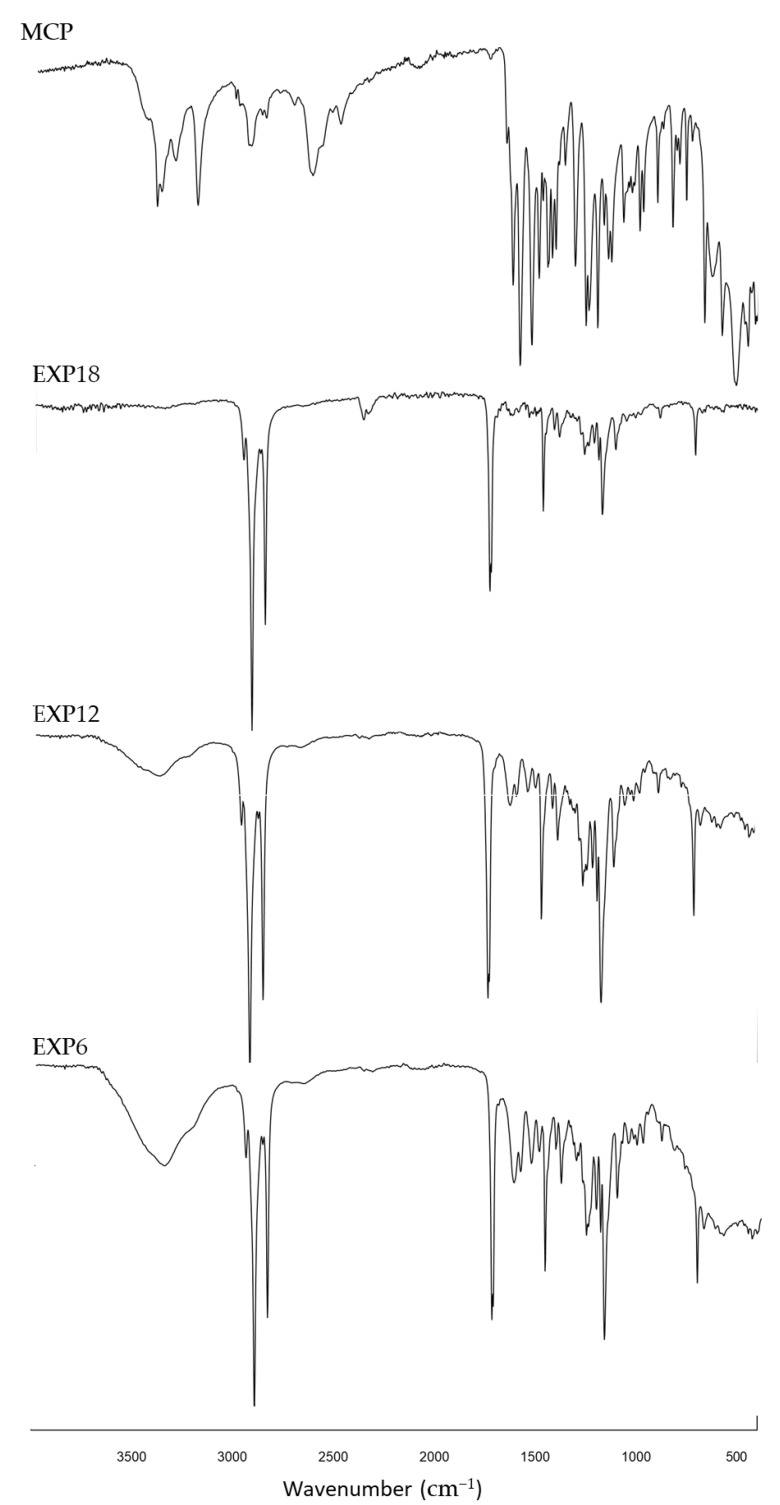
IR spectra of EXP 6 (API load = 22.5%), EXP 12 (API load = 12.5%), EXP 18 (API load = 7.5%) and drug.

In IR spectra of microparticles, the typical signals of SFT at 1471, 1742 and 2911 cm^−1^ were evident and the peaks from 1630 to 1500 nn and between 3350–3400, due to fact that the stretching of primary ammine revealed the presence of MCP [29] (Figure 10). The relative transmittance increased as a function of drug loading, indicating an effective correlation between the intensity of the aforementioned peaks and the presence of the active ingredient in the systems (IR spectra of SFT and drug/excipient physical mixture are available as additional documentation, Figure S4).

### 3.8. X-ray Powder Diffraction (XRPD)

**Figure 11:**
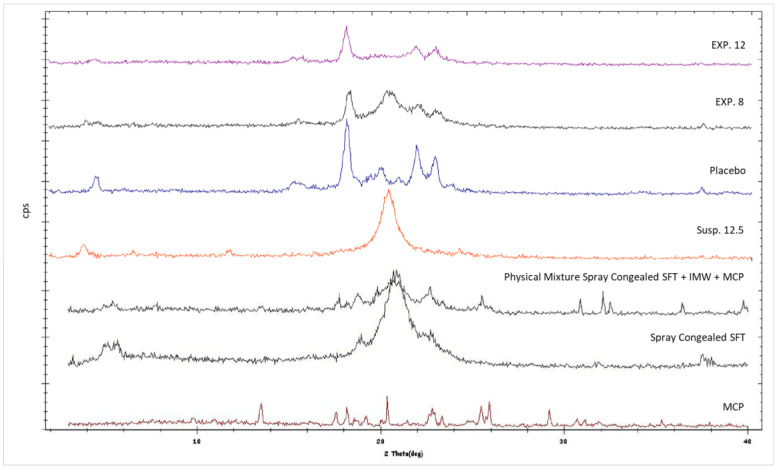
XRPD of EXP 8, EXP 12, drug and references (Placebo = Spray Congealed SFT + IMW + WTR).

XRPD analysis shows that lipid polymorphic transition α-β occurred in a manner depending on the presence of XRPD. This finding is supported by Figure 11, which shows that SFT, after melting and re-solidification, resulted in its α form, the well-known metastable form of fats and triglycerides characterized by type H sub-cell and by XRPD spectra with a single peak [30,31]. This metastable form was also present in the formulations obtained starting from a suspension (see Formulation Susp. 12.5 in Figure 11), where the single peak is easily noticeable. On the contrary, if the lipid was sprayed by the use of the emulsion method (i.e., presence of water), it was possible to note the presence also of the most stable form, indicating that the transition to the latter occurred with a gradual reduction of the intensity of the peak of the α form (see Formulations EXP 8, EXP 12 and Placebo).

It is known that the transition rate can be altered by adding substances and by the nature of these substances. For example, diacylglycerols and sucrose esters stabilized the metastable forms of triglycerides and solid surfactants retarded the polymorphic transitions of tristearin [32,33], while some liquid emulsifiers tended to enhance the transformation [34]. For example, Pattarino et al. [35] found that the presence in the liquid state of medium chain triglycerides (MCT) in gliceriltristearate (GTS) mixtures had a significant impact on the crystallization of GTS stable form, with a promotion and MCT concentration-dependent effect on α/β transition. In the case of SFT, the promotion effect could be ascribed to the presence of water, because only in formulations in which water was used was the α/β transition observable. The water probably acted as a molecular mobility enhancer and led the H sub-cell of the metastable form to easily convert into the triclinic sub-cell packing of the stable form [33]. The α/β conversion is a matter of debate in pharmaceutical fields, with the main focus on understanding if the transition affects and alters the release properties of the SLMps. Recently Bertoni et al. [6] demonstrated that the metastable form of tristearine promoted a faster release of a model drug than the stable one does, suggesting that the difference in release properties between α and β forms can be associated with the most compact arrangement of the β-crystals compared to α-crystals. This was observed when the drug content was high, but based on the data obtained from the experiments, it is challenging to discriminate whether the fat crystal structure or the porosity generated by water has more impact on the API release profile.

XRPD data showed no presence of MCP peaks in all formulations produced by both emulsion and suspension methods. This result suggested that a non-crystalline form of MCP (amorphous) was in microparticles, or the drug was molecularly dispersed in the lipid matrix. If, for the emulsion method, the presence of a non-crystalline form of MCP can be reasonable, due to the initial solubilization of the API in water, in the case of the suspension method, in which no solubilization occurred (there was no water and MCP did not solubilize in lipid), this result was quite unexpected and probably has to be attributable to other events. For example, in literature, it is reported that metoclopramide, when heated in a temperature range from 78 to 105 °C, can undergo several changes such as dehydration, or recrystallization of the anhydrous or amorphous form formation depending on process conditions [36,37]. Based on the statements above, it can be reasonable to assume that the API was in its amorphous form in the suspension method.

## 4. Conclusions

In this paper, the production of SLMp by an unusual technique was proposed and successfully realized. In detail, the melted slurry submitted to the spray congealing process was a W/O emulsion in which the dispersed phase was a water solution of a hydrophilic model drug. The formulative test carried out through a ternary diagram allowed the development of stable emulsions (micro-emulsion or colloidal systems) and suggested many possibilities for developing SLMp with tailored characteristics.

The obtained microparticles presented a spherical shape, particle size distribution between 54–98 µm depending on atomizing pressure used during the production step and 2–5% residual water 4 days after the preparation. In vitro drug release tests highlighted that all the formulations have a reduced release rate compared to the drug alone. These results suggest that spray congealing of a W/O emulsion could be proposed as a good strategy to obtain SLMp with a high loading of a hydrophilic drug and able to control its release rate

The ability of these systems to be processed even if they initially contain a consistent amount of water opens new potential lines of research for developing interesting microparticle delivery systems. Moreover, considering that the presence of water alters the polymorphic behavior of the lipid matrix used in this study, further studies will be carried out to better understand the relationship between the dispersed phase, the lipid crystal lattice and the release behavior of the final product that could have promising application in different fields of pharmaceutical drug delivery.

## Figures and Tables

**Table 1:** Factorial design. Signs “+”, “0” and “-” indicate the levels of factorial design within each factor. API Amount (“+” = 22.5%, “0” = 12.5%, “-” = 7.5%). API:H_2_O (“+” = 1.5:1, “0” = 1:1; “-” = 1:1.5). Pressure Nozzle (“+” = 1.75 bar, “-” = 0.35 bar). For a better comprehension, different colors indicate the different levels.

EXP	API Amount		API: H_2_O		Pressure Nozzle	
	Levels	%	Levels	Ratio	Levels	Bar
1	+	22.5	+	1.5:1	+	1.75
2	+	22.5	+	1.5:1	-	0.35
3	+	22.5	0	1:1	+	1.75
4	+	22.5	0	1:1	-	0.35
5	+	22.5	-	1:1.5	+	1.75
6	+	22.5	-	1:1.5	-	0.35
7	0	12.5	+	1.5:1	+	1.75
8	0	12.5	+	1.5:1	-	0.35
9	0	12.5	0	1:1	+	1.75
10	0	12.5	0	1:1	-	0.35
11	0	12.5	-	1:1.5	+	1.75
12	0	12.5	-	1:1.5	-	0.35
13	-	7.5	+	1.5:1	+	1.75
14	-	7.5	+	1.5:1	-	0.35
15	-	7.5	0	1:1	+	1.75
16	-	7.5	0	1:1	-	0.35
17	-	7.5	-	1:1.5	+	1.75
18	-	7.5	-	1:1.5	-	0.35

**Table 2:** Summary of experimental points and suspensions (Susp) characterizations (Particle size and Residual water; n.d. indicates not available data.

EXP	Particle Size (µm)			Residual Water (*w*/*w* %)		API Recovery (%)
	D10	D50	D90	t0	t4	
1	6.25	21.58	55.41	5.57	1.45	100.3
2	8.73	47.92	87.09	6.98	2.87	97.5
3	5.91	15.25	38.50	12.15	5.83	100.6
4	21.22	53.38	94.95	16.28	4.29	99.9
5	5.76	17.88	54.23	12.28	3.12	105.2
6	31.55	67.16	125.03	16.21	2.11	98.9
7	4.92	20.07	54.77	3.56	2.75	106.9
8	17.77	49.66	85.91	6.36	3.42	91.8
9	6.11	18.60	88.48	8.02	5.42	104.6
10	20.49	60.08	121.50	9.10	3.44	107.1
11	6.41	23.20	60.20	4.60	2.19	107.2
12	18.13	47.78	81.57	8.95	3.29	97.8
13	4.22	16.62	45.59	4.50	2.57	105.5
14	20.43	54.35	93.68	3.87	1.56	99.9
15	7.18	18.54	46.18	3.07	2.03	106.7
16	18.65	53.41	101.90	4.50	2.16	99.2
17	4.09	15.93	45.67	4.36	1.72	97.4
18	16.55	47.23	84.71	4.67	3.63	97.4
Susp 7.5	15.20	50.69	91.11	n.d.	n.d.	n.d.
Susp 12.5	35.57	87.89	161.20	n.d.	n.d.	n.d.
Susp 22.5	20.66	67.92	121.00	n.d.	n.d.	n.d.

## Data Availability

Not applicable.

## Supplementary Materials

The following supporting information can be downloaded at: https://www.mdpi.com/article/10.3390/pharmaceutics14122805/s1, Figure S1: zoom on the first 60 min of the in vitro release profiles of experiments with the same API amount; Figure S2: zoom on the first 60 min of the in vitro release profiles of experiments with the same API:H_2_O ratio; Figure S3: percentages of API in solution after 240 and 1440 min, and after sample heating; Figure S4: IR spectra of SFT and of drug-excipient physical mixture; Table S1: Coefficient estimation for equation 6.
