# Supplementary material for: Solid Lipid Microparticles by Spray Congealing of Water/Oil Emulsion: An Effective/Versatile Loading Strategy for a Highly Soluble Drug
Source: Pharmaceutics. 2022 Dec 14;14(12):2805. doi: 10.3390/pharmaceutics14122805 (PMC9785713; doi:10.3390/pharmaceutics14122805)
Supplement: Supplementary file 1 [file pharmaceutics-14-02805-s001.zip › pharmaceutics-2072754-supplementary.pdf]

ADDITIONAL DOCUMENTATION

Table S1: Coefficient estimation for equation 6

Factor	Coefficient Estimate	95% CI Low	95% CI High
Intercept	31.48	28.29	34.67
A-API Amount	21.95	18.15	25.76
B-API:H_2O	-7.24	-11.05	-3.44
AB	-10.71	-15.24	-6.17

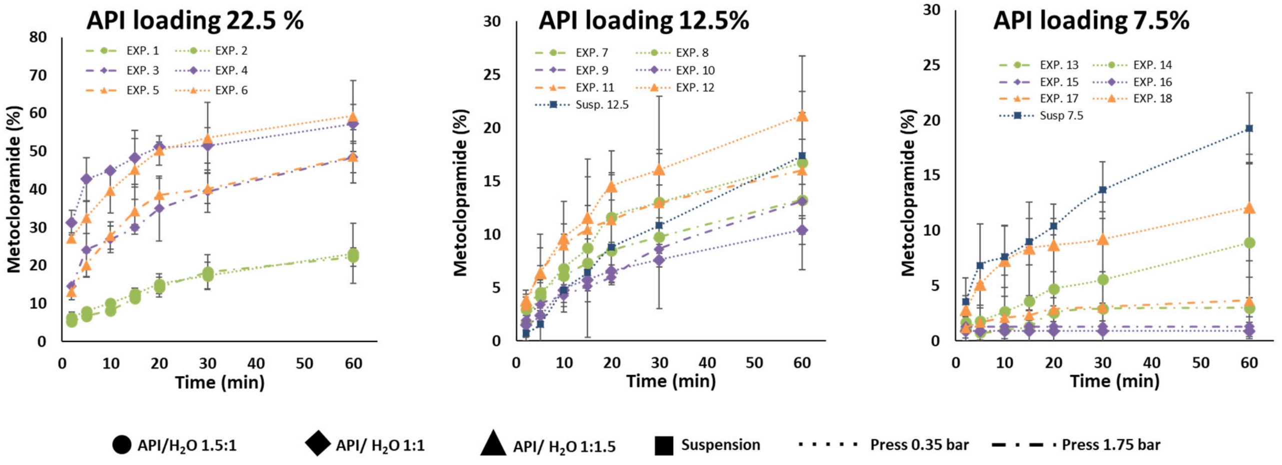

Figure S1: zoom on the first 60 minutes of the *in vitro* release profiles of experiments with the same API amount

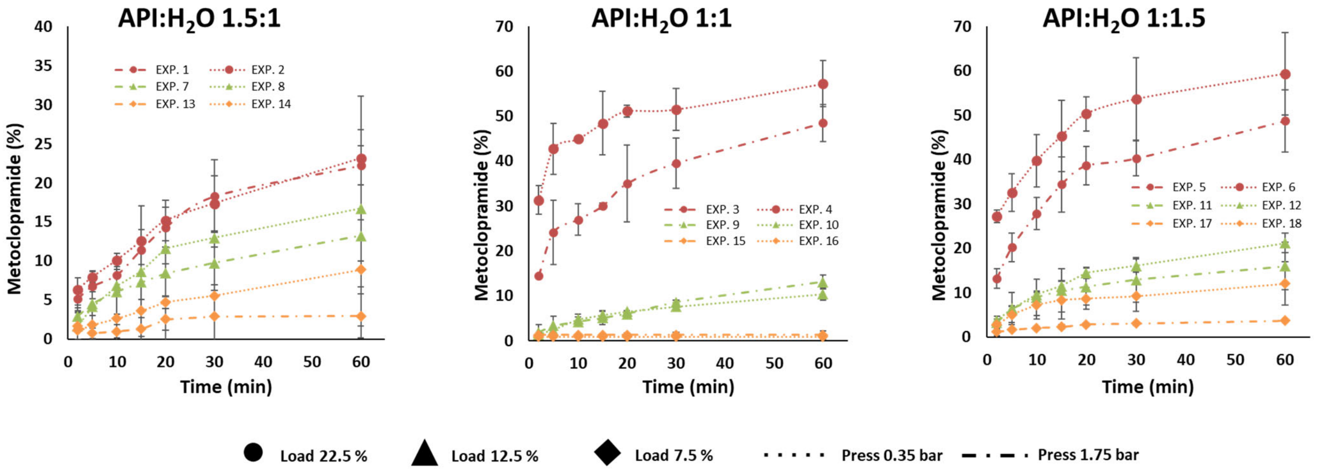

Figure S2: zoom on the first 60 minutes of the *in vitro* release profiles of experiments with the same API:H_2O ratio

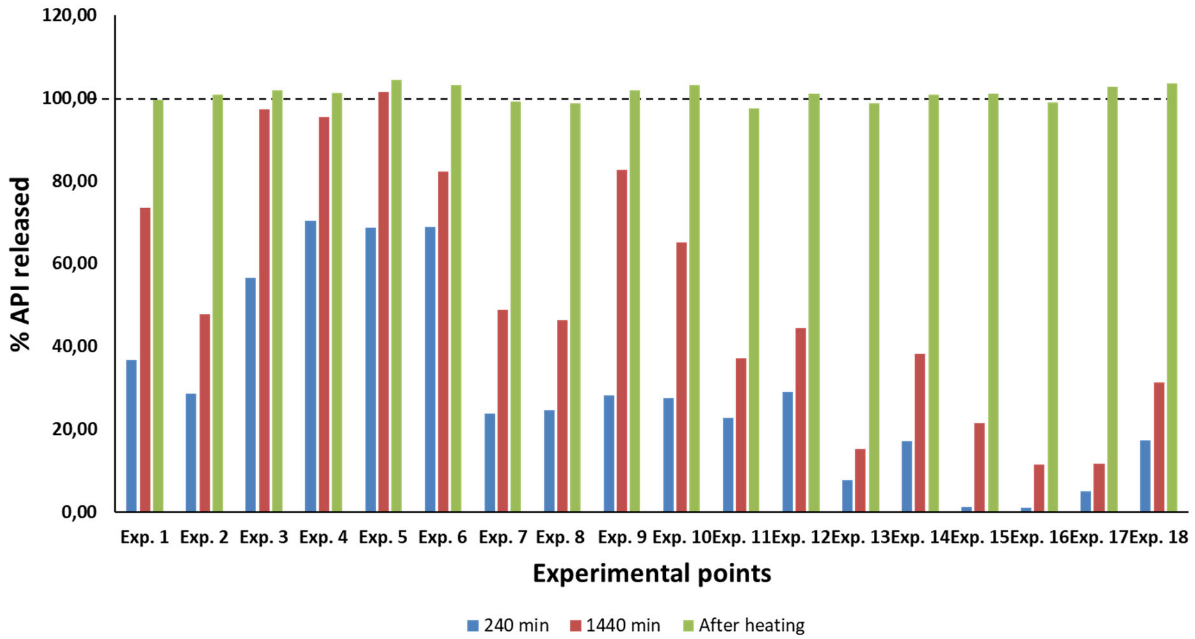

Figure S3: percentages of API in solution after 240 and 1440 min, and after sample heating

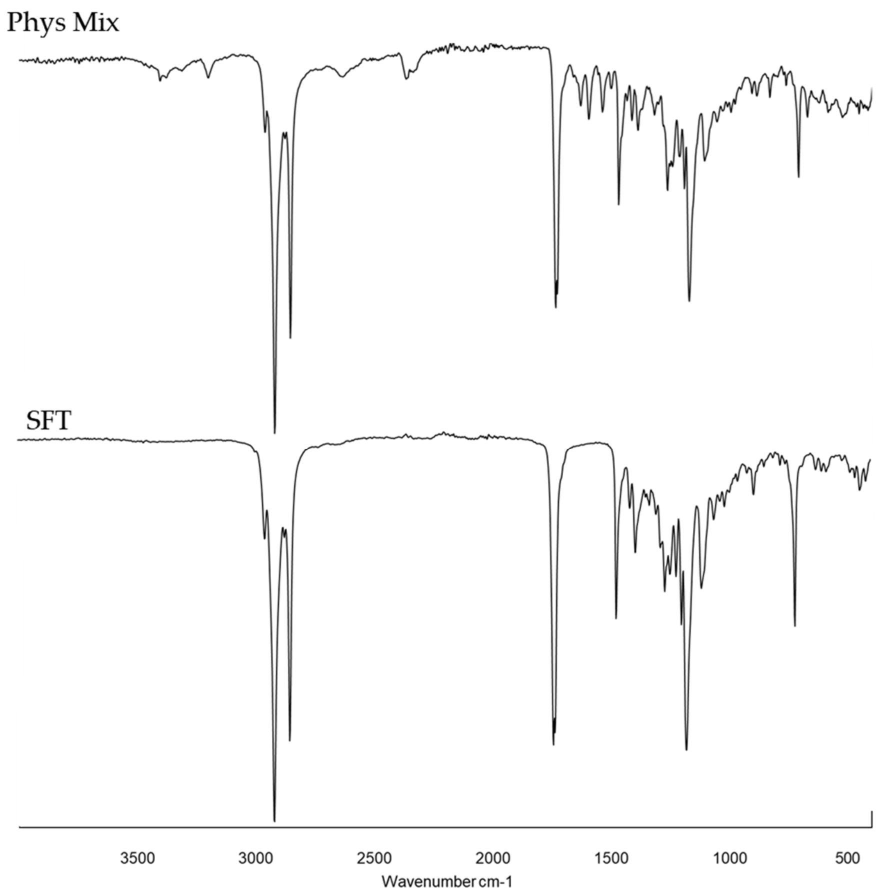

Figure S4: IR spectra of SFT and of drug-excipient physical mixture.
